# Electrochemical Mechanism of Oxidative Dissolution of Silver Nanoparticles in Water: Effect of Size on Electrode Potential and Solubility

**DOI:** 10.3390/nano13131907

**Published:** 2023-06-22

**Authors:** Boris Ershov, Vadim Ershov

**Affiliations:** Frumkin Institute of Physical Chemistry and Electrochemistry, Russian Academy of Science, Leninsky Pr. 31-4, 119071 Moscow, Russia; vadersh@yandex.ru

**Keywords:** nanoparticles, silver, dissolution, solubility, electrochemical mechanism, standard potential, size effect, aggregation

## Abstract

For the first time, an electrochemical mechanism of oxidative dissolution of silver nanoparticles in aqueous solutions is suggested and substantiated. The dissolution is caused by the occurrence of two interrelated electrochemical processes: (1) silver oxidation on a microanode and (2) oxygen reduction on a microcathode. According to the suggested model, the standard electrode potential of a nanoparticle decreases with a decrease in its size, which leads to an increase in the electromotive force of the oxidative dissolution of silver. A proportional dependence of the solubility of nanoparticles on their standard potential is revealed. An empirical equation is derived that relates the solubility of AgNPs to their electrode potential and size. In the course of oxidation, silver nanoparticles undergo aggregation with a gradual increase in the potential to the value characteristic of the bulk metal. This leads to the deceleration and practical cessation of the dissolution.

## 1. Introduction

In the past two to three decades, silver nanoparticles (AgNPs) have been used in industry, medicine, and agriculture [1]. The interest in them is largely due to their antibacterial effect and the possibility of developing medicines based on them, exhibiting the properties of a prolonged-action antibiotic with no allergic effect [2,3,4,5]. However, the downside of the biocidal effect of silver is its toxicity. AgNPs enter the environment in the course of their production and use or after disposal. Uncontrollable spread of silver in nature is dangerous for living bodies and is a real environmental hazard [6]. AgNP migration, aggregation, and dissolution in natural water favor the spread of AgNPs into habitats and enhancement of the toxic effect. Study of these processes and of the relationships of their occurrence is important for evaluation of the environmental risks and ways to prevent them [6,7].

The nanosized state of silver is manifested in nonspecific effects of the dissolution and in their dependence on the size, shape, crystal structure, surface charge, presence of stabilizing additives, and other characteristics of particles [8]. AgNP dissolution with the release of Ag^+^ ions occurs in the presence of air [9,10] and becomes more active with an increase in the solution acidity [10,11,12,13,14,15]. AgNP size is the decisive factor influencing dissolution. The dissolution rate increases with a decrease in the particle size [10,16,17,18,19,20,21]. This trend was attributed to the effect of surface tension [17]. These results are consistent with the finding that AgNP solubility can be estimated from the TEM-derived particle size using the modified Kelvin equation for particles 5−40 nm in diameter. According to Molleman and Hiemstra [22], the release of Ag^+^ is controlled by (1) an equilibrium process with a structural transformation of the surface and (2) a dissolution process with opening of the surface followed by lateral removal of the surface layer. The surface equilibration is size dependent due to a surface Gibbs free energy contribution.

Thus, oxidative dissolution of silver nanoparticles is important from both theoretical and practical standpoints, because it determines the biocidal activity of the particles on the one hand and their toxicity in the habitat on the other hand. Despite significant advances in the study of the solubility of silver nanoparticles and the influence of particle size and various environmental factors on this process, there is no justified rigorous model that would describe the entire set of results obtained and, most importantly, would have the ability to qualitatively and quantitatively predict the expected effect.

Here, we suggest an electrochemical model of AgNP dissolution in water, taking into account the real dependence of their standard electrode potentials on the particle size. In our article, we propose a theoretical model of electrochemical dissolution of silver nanoparticles. To confirm the model, we used experimental data from previously published papers, which also contain a description of experimental research methods. Moreover, we obtained an empirical equation describing the dissolution depending on the size and potential of AgNPs. The data obtained show that a decrease in AgNP potential with a decrease in the particle size is the most probable cause of the observed acceleration in oxidative dissolution.

## 2. Results

### 2.1. Electrochemical Mechanism of the Oxidative Dissolution of Silver Nanoparticles

**Figure 1:**
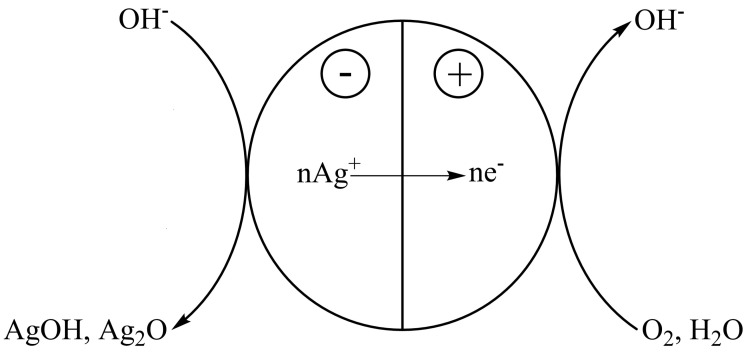
Scheme of the electrochemical dissolution of silver nanoparticles.

The decisive factor that determines the progress of the electrochemical oxidative dissolution of silver is the heterogeneous character of the process involving a high-conductivity solid (metal) and a liquid electrolyte. According to this mechanism of the dissolution (corrosion) [23], two interrelated processes, cathodic and anodic, occur simultaneously on the surface of a solid conductor (silver, in this case) (Figure 1).

As a result of surface heterogeneity, migrating electrodes connected by currents through the conducting metal arise on the nanoparticle surface in contact with an electrolyte. In contact with an aqueous electrolyte containing an oxidant (oxygen, in this case), these voltaic cells favor electrochemical dissolution (oxidation) of silver. The formation of local voltaic couples is favored by differences in the particle shape, presence of oxides and microstructural inclusions on the surface, dislocation outcrops on the surface, and crystal anisotropy. It should be noted that voltaic couples can also arise on the conductor surface because of local heterogeneity of the solution composition (the region with limited oxidant access will act as an anode relative to the region with the free access, which accelerates the electrochemical corrosion) and heterogeneity of the physical action on the surface (temperature, diffusion, etc.).

The electrochemical dissolution of silver can be described by two simultaneously occurring half-reactions (1a) and (1b). On the anode, silver atoms donate electrons, and the ions pass into the solution or form an oxide phase (dissolution):

Ag − e^−^ → Ag^+^, (1a)

Ag^+^ + OH^−^ → AgOH (1b)

Ag + OH^−^ − e^−^ ↔ AgOH (1)

On the cathode, excess electrons interact with the dissolved oxygen, reducing it:

O_2_ + 2H_2_O + 4e^−^ ↔ 4OH^−^ (2)

Acceleration or inhibition of the anodic (or cathodic) process leads to acceleration or inhibition of the cathodic (or, correspondingly, anodic) process. That is the mechanism of the interrelation of the separately occurring processes. At the interface of the conducting phases (AgNPs/aqueous medium, in this case), an electrical double layer (EDL) is formed, and the potential difference arises. The electrode potentials determine the efficiency of the corrosion process. In metal corrosion (oxidative dissolution), equilibrium with the own cations is attained in the electrolyte. The equilibrium potential follows the Nernst equation, and on reaching it, the electrochemical corrosion ceases (equilibrium state). The dissolution cessation can also be caused by kinetic factors such as deceleration of the oxidant supply to the electrode surface and of the product removal from the surface and by the formation of a new phase on the surface, preventing access to the reagents. The silver dissolution involves, most probably, chemical transformation with the formation of a poorly soluble compound or of a stable complex with the present anions (OH^−^, Cl^−^, Br^−^, I^−^, S^2−^, etc.) and/or changes in the EDL of colloids and in their stability, leading to particle aggregation. Therefore, from the thermodynamic standpoint, the process is often irreversible.

The standard electrode potentials E^0^(Ag^+^/Ag) and E^0^(O_2_/OH^−^) for the participants of half-reactions (1) and (2) are 401 and 342 mV versus NHE, respectively [24].

After summation of half-reactions (1) and (2), the reaction of the silver oxidation with oxygen can be described in the form:

O_2_ + 2H_2_O + 4Ag + 4OH^−^ ↔ 4AgOH (or 2Ag_2_O + 2H_2_O) + 4OH^−^ (3)

After cancellations in the left- and right-hand parts of Equation (3), we obtain a simple stoichiometric expression for the silver oxidation with oxygen in water:

O_2_ + 4Ag ↔ 2Ag_2_O (4)

According to a number of papers [10,16,17,19], the Ag^+^ release is a cooperative oxidation process requiring both protons and dissolved O_2_. Silver oxide Ag_2_O dissolves via reaction with protons:

Ag_2_O + 2H^+^ → 2Ag^+^ + H_2_O (5)

Thus, following the suggested mechanism of the oxidation of silver nanoparticles and summing up the reactions of the Ag_2_O formation (reaction (4)) and its subsequent dissolution (reaction (5)), we obtain, in agreement with these papers [10,16,17,19], the following stoichiometric equation of the oxidative dissolution of silver:

1/2O_2_ + 2Ag + 2H^+^ ↔ 2Ag^+^ + H_2_O (6)

### 2.2. Electrode Potentials of Silver Nanoparticles

For AgNP oxidation by the electrochemical mechanism in half-reactions (1) and (2), in accordance with the Nernst equation and Reaction (4), we obtain the following expression for the electromotive force (EMF) ∆E^0^_EMF_:

∆E^0^_EMF_ = [E^0^(O_2_/OH^−^) − E^0^(Ag^+^/Ag)] − 59n^−1^lg[O_2_]^−1^, (7)

where n is the number of electrons exchanged in the redox reaction (four, in this case). In this equation, E^0^(Ag^+^/Ag) is the standard electrode potential of silver, including the potential of nanoparticles of different sizes (reaction 1). For bulk silver, this potential is 342 mV. It could be expected a priori that the potential would depend on the nanoparticle size because the fundamental characteristics significantly change when moving to the nanosized state of a substance. As will be shown below, this assumption is confirmed experimentally. At a constant oxygen concentration, the difference [E^0^(O_2_/OH^−^) − E^0^(Ag^+^/Ag)] is the relative measure of the ∆E^0^_EMF_ of electrochemical oxidative dissolution of silver depending on the particle size. For the bulk metal, the ∆E^0^_EMF_ is 59 mV. The positive value of EMF suggests the thermodynamic possibility of the oxidation of bulk (which should be emphasized) silver. However, previously published results have shown that the standard electrode potential can be changed when the particle size of the electrode reaches the nanometer scale due to its strong surface effect. According to theoretical calculations [25,26,27], the standard electrode potential of metal nanoparticles decreases with a decrease in their size. This conclusion is also confirmed by the results of experimental studies [28,29,30,31]. Redmond et al. [28] studied the electrochemical nature of colloidal silver particles in contact with water. A negative shift in the electrode potential of the particles was found, as well as the fact that small particles are more prone to oxidation than bulk metal. It was shown [29] that the particle size has a significant effect on the standard electrode potential and the thermodynamic properties of electrode reactions. The data of theoretical studies indicate a significant effect of the size of nanoparticles on electrochemical thermodynamics. Of particular interest are the data of Ivanova and Zamborini’s work [30]. These authors carried out voltammetric studies of the oxidation of AgNPs obtained by reduction with citrate and established the experimental dependence of the potential on the particle size (in the range from 8 to 50 nm). The negative anodic current corresponds to the reaction Ag^0^ − e^−^ → Ag^+^. It should be noted that the same reaction is the first step of silver oxidation with the formation of poorly soluble oxide (1a) and is followed by the second step, Ag^+^ + OH^−^ → AgOH (1b). The size significantly affects the electrode potential of AgNPs. For example, for nanoparticles with a size of approximately 10 nm, the shift in the potential of the reaction Ag^0^ − e^−^ → Ag^+^, according to Ivanova and Zamborini [30], is approximately –140 mV. Then, the standard potential of Reaction (1) for such silver nanoparticles will be approximately 202 mV and not 342 mV as for the bulk metal. This means that the ∆E^0^_EMF_ of the electrochemical oxidation of silver nanoparticles of approximately 10 nm size will be equal not to 59 mV as for the bulk metal, but to 199 mV. An increase in the ∆E^0^_EMF_ stimulates the acceleration of the oxidative dissolution of silver. That is, the nanosized state of silver accelerates the oxidative dissolution of the metal in water by the electrochemical mechanism, and the equilibrium of Reaction (3) of silver oxidation is significantly shifted to the right.

**Figure 2:**
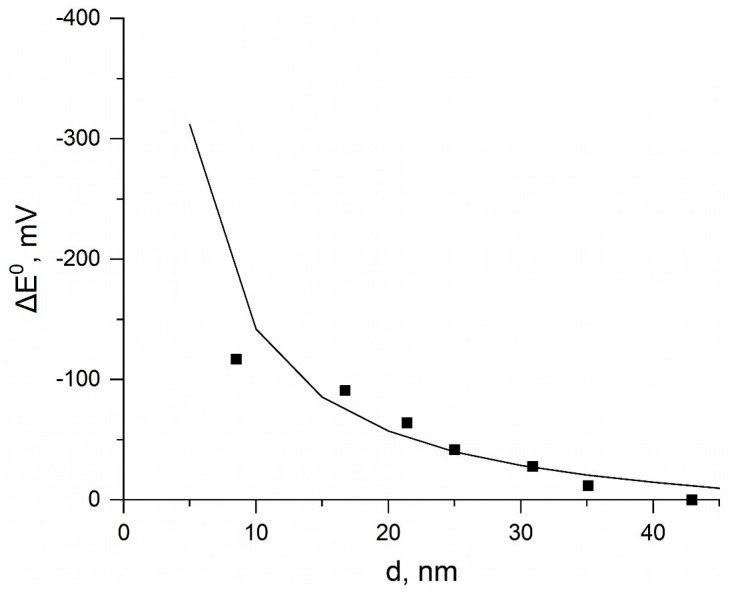
Points: experimental dependence of the shift in potential ΔE_1_ of the reaction Ag − e^–^ → Ag^+^ of AgNP anodic oxidation vs. bulk Ag on the NP diameter [30]. The curve corresponds to the dependence described by the equation ΔE_1_ (mV) = –1750/d + 28. Pearson correlation coefficient r = 0.98247.

To substantiate the electrochemical mechanism of the oxidative dissolution of silver nanoparticles, it was necessary to determine the electrode potentials E^0^(Ag^+^/Ag_NP_) for particles of different sizes. They were calculated from the correlation of the experimental shift in potential of the reaction Ag − e^−^ → Ag^+^ of AgNP anodic oxidation of different sizes d (in the range from 8 to 50 nm) in comparison with the potential of bulk silver [30]. That is, E^0^(Ag^+^/Ag_bulk_) = E^0^(Ag^+^/Ag_NP_) + ∆E_1_ and the numerical values of the shift ∆E_1_ were taken with a negative sign. As we found, the experimental data of the paper cited (Figure 2) can be satisfactorily described by the following equation:

ΔE_1_ (mV) = −(1750 ± 150)/d + (28 ± 8) (8)

**Figure 3:**
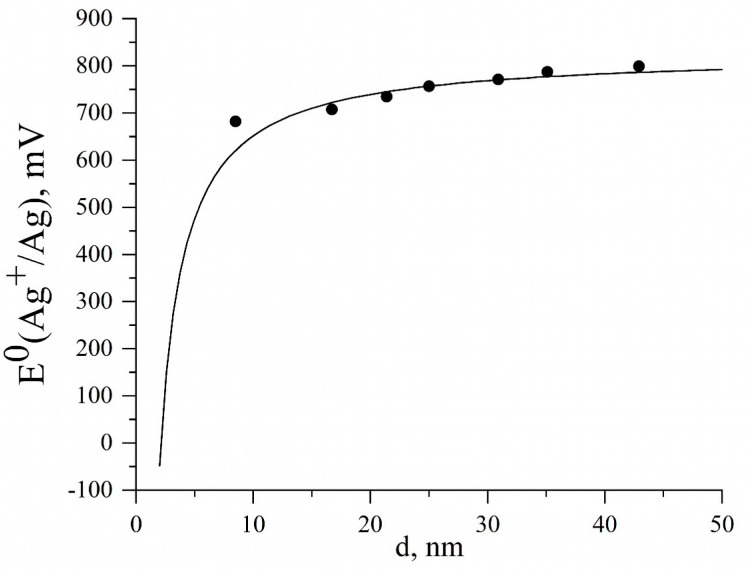
Dependence of electrode potential E^0^(Ag^+^/Ag_NP_) of AgNPs on the size (points) [30]. The curve corresponds to the dependence described by the equation ΔE_1_ (mV) = −1700/d + 28. Pearson correlation coefficient r = 0.98247.

The correlation coefficient between the parameters of the graph in Figure 2 and Figure 3, known as the Pearson correlation coefficient (r), is given in the signature and is equal to 0.98247. Such closeness to the maximum value of one indicates a high degree of correlation between ΔE and d.

The first term in Equation (8) corresponds to the expected theoretical dependence of the potential on the particle size, which has the following form [25,26,27]:

(9) $$\Delta E_{D}=\frac{2g}{r}\frac{V_{M}}{zF^{\prime }}$$

where z is the charge of the metal ions, F is Faraday’s constant, g is the surface stress of the spherical electrode, r is its radius, and v_M_ is the molar volume. The appearance of the additional term in empirical Equation (8) is due to the fact that Ivanova and Zamborini [30] conventionally assumed that ΔE_1_ = 0 at the silver nanoparticle size of 43 nm (because of limited determination accuracy and lack of data for larger particles). Actually, ΔE_1_ tends to be zero at infinite radius. The determination of ΔE_1_ allows calculation of the potential E^0^_1_ and electromotive force ∆E^0^_EMF_ for silver nanoparticles of different sizes. Figure 2 shows the experimental data on the size dependence of ΔE_1_ and the curve describing this dependence. Then, from the known particle size and the dependence of ΔE_1_ on d (or Equation (8)), we determined the corresponding value ΔE_1_ and, after that, calculated E^0^(Ag^+^/Ag_NP_) and ∆E^0^_EMF_.

Next, Figure 3 illustrates the dependence of the electrode potential of silver nanoparticles E^0^(Ag^+^/Ag_NP_) from their size d. The points are derived from the standard silver electrode potential and the shift in potential as E^0^(Ag^+^/Ag_NP_) = E^0^(Ag^+^/Ag_bulk_) − ΔE_1_. The curve in the figure is calculated in a similar way, taking into account that ΔE_1_ is determined by empirical Equation (8). It can be seen that for particles with d ≥ 50 nm, the potential is quite close to E^0^(Ag^+^/Ag_bulk_) (799 mV), and in the size range of 10–20 nm, it noticeably shifts towards lower values and amounts to ~100 mV. Significant changes in the potential are observed for nanoparticles smaller than 5 nm, and for 2 nm or less, the potential is probably already negative. This size range clearly reflects the transition of metal properties from a massive, condensed state to an atomic/molecular state. Note that the potential of the Ag^0^ atom is −1.8 V, while for the Ag_n_ clusters with *n* = 2–6 the potentials are negative [31]. These are particles with pronounced reducing properties. The oligomeric silver clusters, because of their high negative electrochemical potential, are able to reduce water and are instantly oxidized by oxygen [32,33]. Equations (8) and (9) seem to be applicable for silver particles ranging in size from 2 nm to approximately 70 nm.

To confirm the electrochemical model, it is necessary to obtain data on the solubility of nanoparticles with a wide range of sizes and, importantly, in a solution of the same composition. Unfortunately, such data is very limited. In Table 1, we present the data of Peretyazhko et al. [19] on the dissolution of PEGSH-stabilized AgNPs of different sizes in neutral water and acetic acid solution and the corresponding potentials determined by the above-described procedure from the results of Ivanova and Zamborini [30]. Poly(ethylene glycol) methyl ether thiol (PEGSH 5000 Da, NanoCS) was used in that study as nanoparticle stabilizer. The total AgNP concentration at the beginning of dissolution was 74 μM. The degree of Ag^+^ dissolution in % was calculated as [Ag^+^]/[Ag]_total_ × 100%. The data presented illustrate the size effect and the electrode potential on the dissolution of the nanoparticles. As can be seen, with an increase in the silver particle size from ~6 to ~13 nm, the shift in potential ΔE_1_ of the reaction Ag − e^–^ → Ag^+^ of AgNPs anodic oxidation vs. bulk silver (E^0^(Ag^+^/Ag_bulk_)) decreased in the absolute value from –254 to –108 mV. Correspondingly, E^0^(Ag^+^/Ag_NP_) increased from 545 to 691 mV.

**Figure 4:**
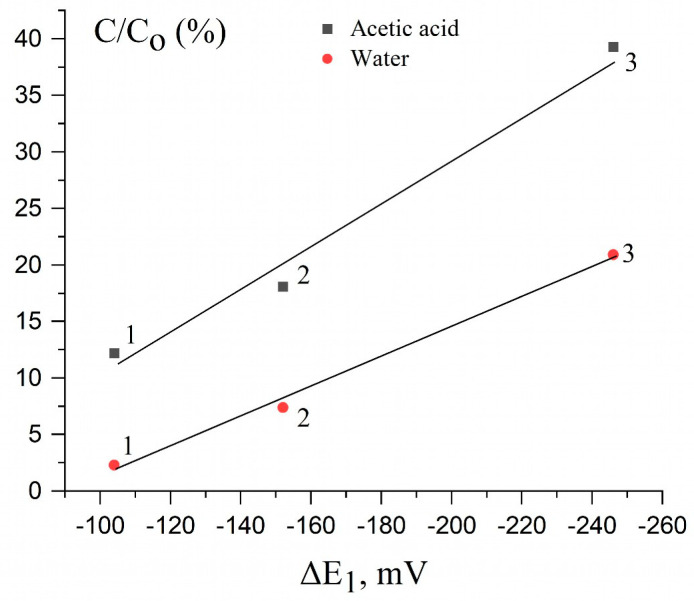
AgNP solubility as a function of the shift in potential ΔE_1_. Particle size, nm: (1) 6.2, (2) 9.2, and (3) 12.9. Initial silver concentration 74 µmol L^–1^. Data of Peretyazhko et al. [19] (AgNP solubility) and Ivanova and Zamborini [30] (shift in potential ΔE_1_ as a function of AgNP size) were used in the calculations. Pearson correlation coefficient r = 0.99154.

That is, the electromotive force the ∆E^0^_EMF_ of oxidative dissolution of nanoparticles decreases with an increase in the size of nanoparticles (313 mV; 281 mV and 167 mV for AgNP sizes of 6.2 nm; 9.2 nm and 12.9 nm, respectively). The parameters of 70-nm nanoparticles are the same as those of the bulk metal, and the ∆E^0^_EMF_ for them is equal to 59 mV. Figure 4 shows the dependence of AgNP solubility on the shift in potential ΔE_1_, based on data from Table 1. As can be seen, the solubility is higher, the larger one is ΔE_1_, and the smaller one is the particle size. In a limited range of nanoparticle sizes (only 6–13 nm), there is linear proportional dependence of the solubility on ΔE_1_, both for neutral water and for an acid solution.

It is known [10,11,16,17,18,19,20,21] that the AgNP dissolution rate increases with a decrease in the particle size. The data of Peretyazhko et al. [19], given in Table 1, illustrate the quantitative relationship between the dissolution rate and the size of PEGSH-stabilized AgNPs. The kinetics is described by a first-order rate equation. In both pure water and an acetic acid solution, the rate constant decreases by a factor of 6–8 with an increase in the particle size from 6 to approximately 13 nm. The effect is due to the change in the electrode potential with particle size. With a decrease in the size, the potential E^0^(Ag^+^/Ag_NP_) decreases compared with that of the bulk metal, E^0^(Ag/Ag_bulk_) (Table 1). As a result, the electromotive force ∆E^0^_EMF_ of the oxidation reaction (3) increases. A comparison of the dissolution rates with the potentials indicates their proportional dependence (Table 1).

**Figure 5:**
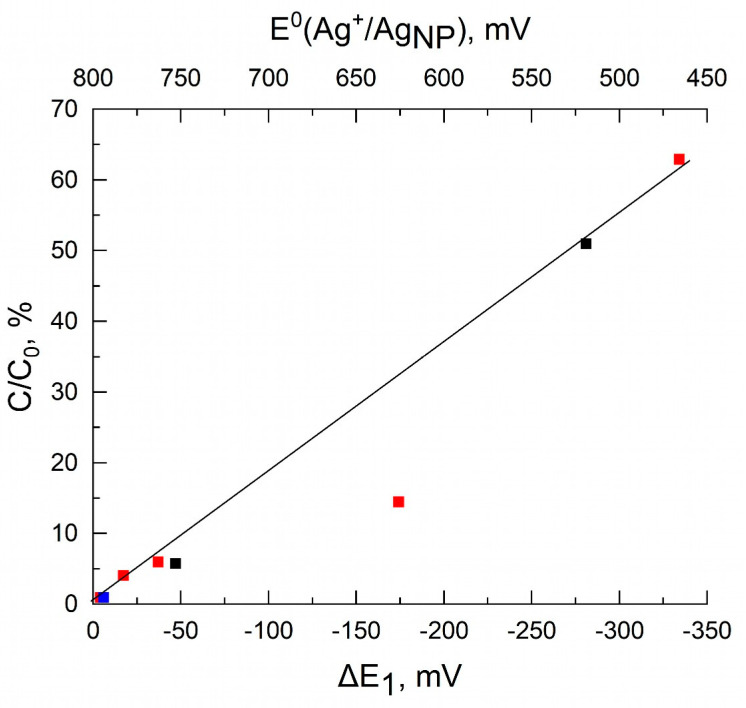
AgNP solubility as a function of shift in potential of ΔE_1_. (1, red) PVP-stabilized AgNPs, size 4.7, 8.4, 26.3, 38.2, and 80 nm; (2, black) gum arabic (GA) stabilized AgNPs, size 5.5 and 22.8 nm; and (3, blue) commercial AgNPs, size 50 nm. Initial silver concentration C_0_ = 47 µmol L^–1^. The calculations were based on the data of Ma et al. [17] (AgNP solubility) and Ivanova and Zamborini [30] (shift in potential ΔE_1_ as a function of AgNP size). Pearson correlation coefficient r = 0.96741.

Figure 5 shows a similar dependence of AgNP dissolution from their electrode potentials built on the basis of data from another study [17], which used a fairly wide set of AgNPs (eight pieces) ranging in size from 4.6 to 80 nm. Polyvinylpyrollidone and Gum arabic were used as stabilizers, and the dissolution was performed in a 1 mmol L^−1^ NaHCO_3_ solution corresponding to the composition of natural water. The dependence of the solubility of AgNPs on both the potential shift ΔE_1_ and the standard electrode potential E^0^(Ag^+^/Ag_NP_) is shown. There is linear correlation between the solubility and the values of ΔE_1_ and E^0^(Ag^+^/Ag_NP_). The dependence is satisfactorily performed both for small particles with a size of 4.6 nm and for 5.4 nm (E^0^ are 447 mV and 503 mV), for which the solubility is 63% and 51%, and for large particles with a size of 26.3 nm and 38.2 nm (E^0^ are 760 mV and 781 mV), for which the solubility is 6% and 4%, respectively. Finally, for very large particles (50 nm and 80 nm) the electrode potentials practically do not differ from the standard electrode potential of bulk silver (799 mV), and the solubility for them is close to zero. Only one point for the 8.4-nm particles is produced from this correlation. The whole set of data shown in Figure 4 and Figure 5 suggests an electrochemical mechanism of the oxidative dissolution of silver nanoparticles in water and aqueous solutions. The smaller one is the particle size, the lower one is its E^0^(Ag^+^/Ag_NP_), and the higher one is the oxidative dissolution in an aqueous medium. The correlation coefficients between the parameters of the graphs in Figure 4 and Figure 5 are equal to 0.99154 and 0.96741, respectively, which indicates a high degree of correlation between solubility and potential. The angle of inclination of the straight lines in Figure 4 and Figure 5, i.e., the coefficients α in Equation 5, turn out to be equal to 0.15 % mV^−1^ and 0.19 % mV^−1^, respectively. Taking into account different methods for obtaining nanoparticles and studying their solubility, there is good agreement in the established quantitative relationship between the solubility of nanoparticles and their potential.

The linear dependence of the solubility of AgNPs on the value of their electrode potential allows us to propose the following empirical equation for determining the concentration of the dissolved metal:

C = C_0_ × α⋅[E^0^(Ag^+^/Ag_bulk_) − E^0^(Ag^+^/Ag_NP_)] = C_0_ × α⋅ΔE_1_, (10)

where α is the coefficient of proportionality, equal to the tangent of the slope of the straight line in Figure 5. Taking Equation (8) into account, which relates the magnitude of shift in potential ΔE_1_ with the size d (nm) of the particle, we obtain the empirical relationship between the oxidation (dissolution) of AgNPs and their size:

C = C_0_ × α⋅[(1750 ± 150)/d + (28 ± 8)] (11)

The resulting equation relates to the specific conditions for carrying out experimental studies in the cited works [17,19]. Obviously, the composition of the medium, the stabilizer, and the concentration of AgNPs play a significant role in oxidative dissolution. However, an empirical approach can be useful in developing a program for predicting and quantifying the release of Ag^+^ into the aquatic environment. According to Equation (8), the standard electrode potential is linearly related to the reciprocal of the average particle size. Empirical Equations 10 and 11 reflect the linear dependence of the solubility on the potential and size of AgNPs, respectively. The empirical coefficient α takes into account the influence of the environment (stabilizing additive, pH, ion concentration, and oxygen content, etc.). Changing the shape of NPs from spherical to another shape changes the structure of the galvanic cell, which, apparently, can change the form of the dependence of dissolution on the potential.

### 2.3. Aggregation of Silver Nanoparticles

Thus, the standard electrode potential of nanoparticles E^0^(Ag^+^/Ag_NP_), which depends on the particle size, determines the efficiency of the dissolution of the metal. When a nanoparticle is oxidized, its physicochemical state and DEL structure change, which reduces its stability and leads to agglomeration and aggregation. The changes are associated with the oxidation of the AgNP surface and an increase in the positive charge of the metal aggregate. As a result, the density of potential-determining ions increases and the diffuse layer is compressed, which contributes to the mutual approach, agglomeration, and aggregation of colloidal particles. This conclusion is confirmed by analysis of the localized surface plasmon resonance (LSPR) absorption of deaerated hydrosol nanoparticles stabilized with carbonate ions [34,35,36]. In contact with air, the LSPR band is red-shifted, and additional absorption caused by the light scattering with nanoparticle agglomerates appears in this region. The TEM data show that the separated nanoparticles in the course of oxidative dissolution agglomerate and form chains of bonded particles [34]. The DLS data also confirm the aggregation of silver colloids upon oxidation. For example, according to Hedberg [9], oxidation of silver nanoparticles of 20, 40, and 80 nm size, obtained in a citrate medium, is accompanied by a sharp increase in their hydrodynamic size, suggesting agglomeration and aggregation. A fast process of aggregation of small AgNPs introduced into water by thermal evaporation of Ag was observed in the work of [28]. It was assumed that the particles aggregate according to the electrochemical mechanism of Ostwald maturation, which is determined by the size dependence of the work function and the standard electrode potential of nanoparticles of different sizes.

The aggregation of AgNPs, in our opinion, is the main reason for the kinetic stopping of silver dissolution. This is indicated by the correlation between the processes of dissolution and aggregation of particles. An increase in the size of the aggregates leads to an increase in their electrode potentials up to reaching the potential of bulk silver. This leads to deceleration and then to practical cessation of the dissolution. The data of Table 1 and Figure 4 show that large AgNPs have a very low dissolution rate. This conclusion is confirmed by the data of Chen et al. [37], who have shown that the anodic oxidation of silver on the level of separate nanoparticles leads to their aggregation on gold microelectrodes. The particle diameter and degree of aggregation significantly influenced the potential. As a result of aggregation, small nanoparticles acquired the potential characteristics of the bulk metal, i.e., ΔE_1_ in the course of aggregation tended to zero. As expected, this was accompanied by a decrease in the dissolution state and attainment of the pseudo-equilibrium. The latter fact is also probably favored by the formation on the metal surface of the oxide and other protecting compounds. This phenomenon is also common in nanoparticles of other metals, primarily gold [38,39,40,41]. In particular, as shown for gold [38], the oxidation potential of the aggregated AuNPs of 4 and 15 nm diameters is shifted toward the positive side by 230 and 180 mV, respectively. The shift depends on the degree of aggregation, which was controlled by pH and time. The UV–Vis spectra of the solution and the SEM images of the electrodes demonstrate aggregation of the 4- and 15-nm particles. On the other hand, the oxidation potential does not change at all for the aggregated AuNPs ≥50 nm in diameter. That is, a cluster of small nanoparticles acquires a potential characteristic of bulk gold. Separate 50-nm particles, in turn, can be considered as the bulk metal. It is emphasized [41] that the oxidation stability of aggregated gold is very sensitive to details of the aggregate structure. For example, aggregates induced with citric acid are three-dimensional with strongly coalescent AuNP–AuNP contacts, whereas aggregates induced with tetrakis(hydroxymethyl)phosphonium chloride (THPC) are linear or two-dimensional with the distance between AuNPs of ~1 nm. Strong AuNP–AuNP contacts make the aggregates similar to bulk gold in the characteristics.

### 2.4. Environmental Significance

Uncontrollable spread of nanosilver is a real environmental hazard. Dissolution is one of the main processes that controls AgNP behavior in aquatic systems. The electrochemical model of the oxidative dissolution of silver nanoparticles in water, developed in this study, established a correlation between the particle size and its fundamental characteristic, standard electrode potential, which exerts a decisive effect on the efficiency and kinetics of AgNP dissolution. The smaller the particle size, the more negative the electrode potential and the higher the rate of its oxidation. The agglomeration of nanoparticles acquires the electrode potential of bulk silver and its inherent low dissolution rate. An empirical equation is derived that relates the solubility of NPs to their electrode potential and size.

## 3. Conclusions

Our study demonstrated that the oxidative dissolution of silver nanoparticles in aqueous solutions occurs, most probably, by an electrochemical mechanism. The fraction of the dissolved metal and the rate of release of the Ag^+^ ions into the solution increase with a decrease in AgNP size. This is caused by a decrease in the standard electrode potential of the reaction Ag − e^−^ → Ag^+^ which, in turn, increases the electromotive force of the silver oxidation with oxygen. In the course of oxidation, AgNPs undergo aggregation with a gradual increase in E^0^(Ag^+^/Ag_NP_) to the value characteristic of the bulk metal. This leads to deceleration and then to practical cessation of the dissolution. This is convincingly shown by Peretyazhko et al., who reported that the extent of dissolution C/C_0_ after 80-day incubation in water decreased in the order AgNPs_6 nm > AgNPs_9 nm > AgNPs_13 nm > AgNPs_70 nm and was equal to 20.9, 7.4, 2.3%, and virtually 0, respectively (Figure 3). Similar dependence of the solubility on the size particle was observed by Ma et al.: AgPVP_5 nm > AgPVP_8 nm > AgPVP_25–38 nm, 5.4, 1.0, and 0.27 mg L^–1^, respectively, whereas for the bulk metal (thin silver plate) the solubility was 0.009 mg L^–1^. That is, the nanoparticle aggregation in the course of oxidative dissolution is accompanied by an increase in the standard electrode potential. This leads to deceleration of the oxidative dissolution. An electrochemical model of the dissolution of silver nanoparticles in water depending on their size and electrode potential was proposed and substantiated for the first time. The developed approach may be promising for describing the solubility of other metals in the nanosized state in an aqueous medium.

## Figures and Tables

**Table 1:** Solubility and electrode potentials of silver nanoparticles of different sizes.

Size, nm	Water		Acetic Acid, 0.05 mol L^–1^		ΔE_1_, mV	E_0_(Ag^+^/AgNP), mV
	[Ag^+^] Total, μM	K × 10^−5^, s^−1^	[Ag^+^] Total, μM	K × 10^−5^, s^−1^		
6.2 ± 1.6	15.5	1.07	29.1	1.43	−254	545
9.2 ± 3.0	5.5	0.37	13.4	0.56	−162	637
12.9 ± 3.5	1.7	0.13	9.0	0.25	−108	691
70.5 ± 12	-	-	4.7	0.21	~0	~799

## Data Availability

Data sharing not applicable.
